# Editorial: Dietary Supplements, Botanicals and Herbs at the Interface of Food and Medicine

**DOI:** 10.3389/fphar.2022.899499

**Published:** 2022-07-04

**Authors:** Alessandra Durazzo, Massimo Lucarini, Michael Heinrich

**Affiliations:** ^1^ Research Centre for Food and Nutrition, Council for Agricultural Research and Economics, Rome, Italy; ^2^ School of Pharmacy, Faculty of Life Sciences, University College London, London, United Kingdom

**Keywords:** dietary supplements, nutraceuticals, dietary supplements labeling, chemometrics, dietary supplement databases, metrology, control quality, food

## Introduction

The Research Topic “Dietary Supplements, Botanicals and Herbs at The Interface of Food and Medicine” provide a detailed and up to date picture on a key interdisciplinary field.

The focus of this Research Topic has been the relationship between dietary supplements (according to FDA definition)/food supplements (according to EFSA definition), botanicals, herbal medicines, herbal medicinal products and, foods with potential beneficial properties from a global and interdisciplinary perspective with the aim of understanding their potential health benefits. In order to simplify the discussion in the context of the complex terminology, herbal remedies, herbal drugs, herbal medicinal products, herbal medicines, botanical drugs are all labelled as botanicals, a term commonly linked to the framework in the USA, but now also used, for example, in Europe (EFSA Scientific Committee, 2009; European Food safety Authority, 2012; U.S. Department of Health and Human Services Food and Drug Administration Center for Drug Evaluation and Research (CDER), 2016; ^1^).

The use of plants in new formulations of dietary supplements was explored.

The beneficial properties of medicinal plants (often having a restricted regional uses) trigger interest in the possibility of developing novel nutraceutical botanical supplements formulations, which can help to support health conditions reducing the need for pharmacological interventions, in particular for individuals who do not qualify for conventional drug-based treatment.

### The Areas Explored

i) Interdisciplinary approaches using emerging and innovative techniques with chemometrics on dietary supplements, the production of standardized formulations, authentication of individual plants, monitoring of the quality of herbs and plants used for botanical supplements, detection of adulteration or contamination of herbs;
ii) studies on the description and use of bioactive compounds i.e., antioxidants in medicinal plants as well as on physiological mechanism and bioaccessibility of compounds;
iii) the application of nanotechnologies for developing novel plant-based products with improved bioavailability, solubility, and efficacy;
iv) studies of medicinal plants using “large data” e.g., extracted from healthcare or other databases e.g., dietary supplement ones, including the application of classification systems, harmonization, and coding procedures;
v) investigations on the functional/nutraceutical characteristics of foods in order to integrate intrinsic nutritional properties;
vi) the use of food waste as a sustainable alternative source of biologically active compounds for botanicals.

The papers published cover an exciting range of themes within this fast evolving area of research.

The strict linkage of territory, medicinal plants and health was underlined in this Research Topic. For instance, Mudau et al. presented a systematic review of the potential of South African neglected and underutilized crops as food and herbal medicinal crops. Vujicic and Cohall reported the knowledge, attitudes and practices on the use of botanical medicines in a rural Caribbean territory. Ojha et al. described the traditional dietary knowledge of a marginal hill community in the central Himalaya, with particular focus on the implications for food, nutrition, and medicinal security. Cordero et al. reported the ethnobotanical documentation of medicinal plants used by the indigenous Panay Bukidnon in Lambunao, Iloilo, Philippines. Okagu et al. provided a stimulating review of *Zanthoxylum* species and their traditional uses, phytochemistry and pharmacology in relation to cancer, infectious diseases and sickle cell anemia. Wu et al. reviewed the ethnopharmacological understanding of *Houttuynia cordata* Thunb. Kasali et al. presented a critical review of ethnopharmacology and bioactivity data of antidiabetic medicinal plants used in Democratic Republic of Congo. Belhouala and Benarba presented a multiregional ethnobotanical study of medicinal plants used by traditional healers in Algeria. Brendler1 and Abdel-Tawab reviewed the features of Buchu (*Agathosma betulina* (P.J.Bergius) Pillans and *Agathosma crenulata* (L.) Pillans).

Research on bioactive metabolites and beneficial properties and effects of medicinal plants is another core focus. Rahim et al. studied the phytochemical analysis, antioxidant and bone anabolic effects of *Blainvillea acmella* (L.) Philipson. Otari et al. reported the phytochemistry of two unexplored endemic medicinal plants of India, *Barleria terminalis* Nees and *Calacanthus grandiflorus* (Dalzell) Radlk. Divyashri et al. showed experimental evidence of neuroprotective potential of non-digestible oligosaccharides. Sadgrove et al. described the pharmacology of natural volatiles and essential oils in food, therapy, and disease prophylaxis.

An overview of meta-analyses of clinical trials of medicinal plants used for glycaemic control in type 2 diabetes is given by Willcox et al. Razali et al. studied *in vitro* anticancer activity of *Neolamarckia cadamba* (Roxb.) Bosser leaf extract and its metabolite profile. Kudera et al. reported on selective *in vitro* antibacterial and antiproliferative effects of ethanolic extracts from Cambodian and Philippine plants used in local medical system for diarrhea treatment.

Zheng et al. reported how the artesunate combined with Metformin ameliorate on diabetes-induced xerostomia by mitigating superior salivatory nucleus and salivary glands injury in type 2 diabetic rats *via* the PI3K/AKT pathway. Mohanty et al. described the ameliorative effects of dietary ellagic acid against severe malaria pathogenesis by reducing cytokine storms and oxidative stress. Rahmi et al. reported how the extracts of *Andrographis paniculata* (Burm.f.) Nees leaves exert anti-gout effects by lowering uric acid levels and reducing monosodium urate cystal-induced inflammation.

Moving towards the field of innovative procedures for medicinal plants and herbs research in quality control area, the international perspective by Durazzo et al. addresses analytical challenges and metrological approaches to ensuring dietary supplement quality. An example is given by Xu et al. that described the authentication of three source spices of *Arnebiae Radix -Arnebia decumbens* (Vent.) Coss. et Kralik*, Arnebia euchroma* (Royle ex Benth.) I.M.Johnst. and *Arnebia guttata* Bunge*-*using DNA barcoding and HPLC. Jiang et al. showed a value chain perspective for the quality monitoring of *Cistanche deserticola* Ma.

Schreiner et al. investigated 68 powdered plant extracts (botanicals) which are added to food products in food industry throughout 8-dimensional hyphenation of normal-phase high-performance thin-layer chromatography with multi-imaging by ultraviolet, visible and fluorescence light detection as well as effect-directed assay and heart-cut of the bioactive zone to orthogonal reversed-phase high-performance liquid chromato-graphy−photodiode array detection−heated electrospray ionization mass spectrometry: the array of 1,292 profiles (68 samples × 19 detections) showed the versatile bioactivity potential of natural food.

One particular focus is the interface between food and medicine. *Prunus mira* Koehne in Sichuan (PR China) is the focus of a review covering its history, distribution, modern application and ethnobotanical investigation (Zhang et al.; Martínez-Francés et al. analysed the medicinal plants in traditional herbal wines and liquors in the east of Spain and the Balearic Islands. Asdaq et al. reported the potential benefits of using garlic oil and its active constituent, dially disulphide, in combination with carvedilol in ameliorating isoprenaline-induced cardiac damage in rats. Otunola et al. reviewed the properties of culinary spices in food and medicine, with focus on *Syzygium aromaticum* (L.) Merr. and L. M. Perry [Myrtaceae].

Iñiguez-Luna et al. studied the natural bioactive compounds of *Sechium* spp. P. Br (chayotes)for therapeutic and nutraceutical supplements. Li et al. reviewed the promising traditional uses, pharmacological effects, aspects, and potential applications *Citrullus colocynthis* (L.) Schrad (Bitter Apple Fruit). Attah et al. studied effect of extracts of moringa oleifera seed on some reproductive parameters, hepatic and renal histology.

Chao et al. presented an ethnobotanical survey on bitter tea in Taiwan.

Krepkova et al. described valuable hepatoprotective plants i.e. milk thistle, artichoke, and chicory and the use of their waste products as rich sources of active components. Alyahya et al. reported the quantification of chlorogenic acid and vanillin from coffee peel extract and its effect on α-Amylase activity, immunoregulation, mitochondrial oxidative stress, and tumor suppressor gene expression levels in H_2_O_2_-induced human mesenchymal stem cells.

Bilal et al. reviewed the nutritional applications, beneficial health aspects of olive oil and its prospective application in poultry production. Oshiomame Unuofin et al. reviewed nutritional and pharmacological applications of ginger from farmyard to town.

Lastly, the development carrot nutraceutical products as an alternative supplement for the prevention of nutritional diseases Riyaz et al.

The research topic has highlighted the diversity of approaches to understand plants which may be a food or a medicine, depending on the context, regulatory status and the interpretation of the evidence. The articles provide fascinating spectrum of perspectives on this important them, relevant for human health and one of the long-standing challenges in ethnopharmacology. They provide not only new insights into specific topics, but also advance our Frontier in research at this interface, which does require considerable more research related to the health benefits and their limitations of such products.

## Footnotes

1
https://ods.od.nih.gov/factsheets/list-Botanicals/.
